# RegPhos 2.0: an updated resource to explore protein kinase–substrate phosphorylation networks in mammals

**DOI:** 10.1093/database/bau034

**Published:** 2014-04-25

**Authors:** Kai-Yao Huang, Hsin-Yi Wu, Yi-Ju Chen, Cheng-Tsung Lu, Min-Gang Su, Yun-Chung Hsieh, Chih-Ming Tsai, Kuo-I Lin, Hsien-Da Huang, Tzong-Yi Lee, Yu-Ju Chen

**Affiliations:** ^1^Department of Computer Science and Engineering, Yuan Ze University, Taoyuan 320, Taiwan, ^2^Institute of Chemistry, Academia Sinica, Taipei 115, Taiwan, ^3^Genomics Research Center, Academia Sinica, Taipei 115, Taiwan, ^4^Institute of Bioinformatics and Systems Biology, National Chiao Tung University, Hsin-Chu 300, Taiwan and ^5^Department of Biological Science and Technology, National Chiao Tung University, Hsin-Chu 300, Taiwan

## Abstract

Protein phosphorylation catalyzed by kinases plays crucial roles in regulating a variety of intracellular processes. Owing to an increasing number of *in vivo* phosphorylation sites that have been identified by mass spectrometry (MS)-based proteomics, the RegPhos, available online at http://csb.cse.yzu.edu.tw/RegPhos2/, was developed to explore protein phosphorylation networks in human. In this update, we not only enhance the data content in human but also investigate kinase–substrate phosphorylation networks in mouse and rat. The experimentally validated phosphorylation sites as well as their catalytic kinases were extracted from public resources, and MS/MS phosphopeptides were manually curated from research articles. RegPhos 2.0 aims to provide a more comprehensive view of intracellular signaling networks by integrating the information of metabolic pathways and protein–protein interactions. A case study shows that analyzing the phosphoproteome profile of time-dependent cell activation obtained from Liquid chromatography-mass spectrometry (LC-MS/MS) analysis, the RegPhos deciphered not only the consistent scheme in B cell receptor (BCR) signaling pathway but also novel regulatory molecules that may involve in it. With an attempt to help users efficiently identify the candidate biomarkers in cancers, 30 microarray experiments, including 39 cancerous versus normal cells, were analyzed for detecting cancer-specific expressed genes coding for kinases and their substrates. Furthermore, this update features an improved web interface to facilitate convenient access to the exploration of phosphorylation networks for a group of genes/proteins.

**Database URL**: http://csb.cse.yzu.edu.tw/RegPhos2/

## Introduction

Protein phosphorylation, which is an important and reversible mechanism in posttranslational modifications (PTMs), is involved in many essential cellular processes including transcriptional regulation, metabolic pathways, cell growth, apoptosis, differentiation, and ions/molecules transport (1). In addition, protein phosphorylation plays essential regulatory roles in intracellular signal transduction, which transmits information from the cell surface to the nucleus, where they ultimately effect transcriptional changes (2, 3). The phosphorylation at serine, threonine and tyrosine residues of eukaryotic proteins are added by serine/threonine and tyrosine kinase families. It has been estimated that one-third to one-half of all proteins in a eukaryotic cell are phosphorylated (4). With the high-throughput of mass spectrometry (MS)-based proteomics in identifying *in vivo* or *in vitro* phosphorylation sites, a variety of databases have been developed to accumulate experimentally verified phosphorylation sites with catalytic kinases, including Phospho.ELM (5), PhosphoSitePlus (6), Phosphorylation Site Database (7), PHOSIDA (8) and PhosPhAt (9). Additionally, the PhosphoGRID (10) is a new database of experimentally verified *in vivo* protein phosphorylation sites from the budding yeast *Saccharomyces cerevisiae*. The Phospho3D (11) is a database containing 3D structures of phosphorylation sites. The PhosphoPOINT (12) provides a robust annotation for kinases, downstream substrates and their interacting phosphoproteins, which could enhance the functional characterization of kinome-mediated signaling. Because a large number of protein phosphorylation sites were identified without the annotation of catalytic kinases, various approaches have been proposed to computationally reveal the kinase-specific phosphorylation sites based on the linear motifs of substrate residues (13–19).

The human kinome has been identified by Manning *et al.* in 2002 (20), which provides a starting point for studying protein phosphorylation networks. A previous work has developed a computational approach for generating static models of signal transduction networks by using protein-interaction maps generated from large-scale two-hybrid screens and DNA microarrays expression profiles (3). Although various methods were proposed to model signaling networks (21–25), the experimental data need to be combined with system biology analysis, which maps large-scale phosphoproteome data sets to signaling networks (26). Recently, a new method has been proposed to integrate physical and functional aspects of phosphorylation network together with the transcription network in *S. cerevisiae*, which demonstrated that different network motifs are involved in these networks (27). Furthermore, a new strategy called CEASAR, based on functional protein microarrays and bioinformatics, has been developed to construct a high-resolution map of phosphorylation networks that connects 230 kinases to 2591 phosphorylation sites in 652 substrates (28).

Although MS/MS phosphoproteome data have enabled the large-scale mapping of protein phosphorylation sites (29), a full understanding of the landscape of intracellular signaling networks remains a major challenge in cellular biology. Therefore, RegPhos has been proposed to integrate experimentally verified protein phosphorylation and protein–protein interaction (PPI) data for constructing the intracellular phosphorylation networks, starting from receptor tyrosine kinases to substrate proteins or transcription factors (TFs) in nucleus, based on Breadth-First Search algorithm (30). In version 2.0 of RegPhos, we not only enhance the data content in human but also investigate the kinase–substrate phosphorylation networks in mouse and rat. This update aims to provide a more comprehensive view of intracellular signaling networks by integrating the experimentally confirmed kinase–substrate phosphorylations, metabolic pathways and PPIs. To validate the utility of RegPhos, this work integrated the quantitative time-coursed phosphoproteomic data to verify the expression profiles of phosphoproteins in the newly discovered phosphorylation networks associated with B cell receptor (BCR) signaling pathway, which functioned as a model study in this analysis. Linking by protein tyrosine kinase Syk, a critical molecule in immune system, it is biologically important to understand the regulation and function of naïve and activated mast cells for cross-talking of B, T or other immune cells (31). Although the BCR signaling has been studied for several decades and many key molecules and pathways were depicted (32), additional work in this area is to define the interconnections among membrane, cytoplasmic and nuclear events. Further system-wide characterization of the signaling cascades mediated by Syk, BCR signaling or global immune response will broaden our understanding of diseases resulted from immunodeficiency or autoimmune disorders, which may provide clues for development of effective therapeutic strategies. Although elucidation of immune signaling is a daunting challenge, we expect that it can be overcome with the aid of bioinformatics and proteome analysis.

Furthermore, a previous study has reported that around half of kinome is disease- or cancer-related by chromosomal mapping (20). To help users identify the candidate biomarkers of kinase-associated genes in cancers, the microarray expression data containing 39 cancerous versus normal cells is integrated in this update. Finally, RegPhos 2.0 features an improved web interface to facilitate the access to the informative resource, which allows users to input a group of proteins/genes and the system will efficiently return the protein phosphorylation networks associated with three network models, such as PPIs, subcellular localization and metabolic pathway.

## Improvements

The highlighted improvements and advances in RegPhos 2.0 were presented in Supplementary Figure S1 including data enhancement in mammals, network construction using KEGG pathways and PPIs, network validation with time-dependent phosphoproteome profiling, as well as the expression analysis of kinase and substrate genes in 39 cancer type. In addition, the web interface was redesigned and enhanced to facilitate the study of protein phosphorylation networks. This update not only integrated the experimental phosphorylation data from public resources and research articles but also integrated the quantitative time-resolved phosphoproteomic profile obtained from LC-MS/MS analysis. The details of each improved process were depicted as follows.

### Data enhancement in human, mouse and rat

**Figure 1.**
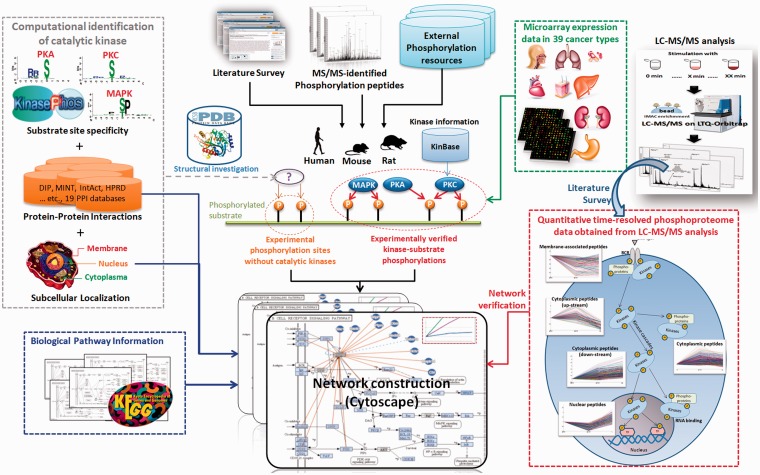
The system flow of RegPhos 2.0.

Figure 1 presented the system flow of RegPhos 2.0. The experimentally verified phosphorylation sites were mainly extracted from dbPTM (33, 34), which has integrated six phosphorylation-associated resources, Phospho.ELM (5), PhosphoSitePlus (6), PHOSIDA (35), SysPTM (36), HPRD (37) and UniProtKB/Swiss-Prot (38). Supplementary Table S1 showed the data statistics of each integrated resources. In this update, we not only enhanced the data content of phosphorylation in human but also integrated the experimentally verified phosphorylation sites as well as the catalytic kinases in mouse and rat. Owing to an emerging evidence of MS/MS-based proteomics in identifying phosphorylation sites, the site-specific phosphoproteome data sets were manually extracted from approximately 200 MS/MS-associated research articles using a text mining approach (39). All of the phosphorylation instances, collected from a variety of heterogeneous resources, were mapped to the protein entries of UniProtKB and removed the redundant data by sequence identity. Additionally, to unify the kinase names among a variety of phosphorylation-associated resources that contain various names for a kinase, the information of human (20) and mouse (40) kinases was referred to the annotations in KinBase. According to the annotations of kinase family and subfamily in KinBase, a total of 518 human and 540 mouse kinases were categorized into 221 and 195 kinase families, respectively. However, the annotation of rat kinome is not included in KinBase. Owing to the high sequence homology between mouse and rat, the protein sequences of 540 mouse kinases were used to identify the orthologous kinases in rat, which has identified 306 kinases.

### Network construction using KEGG pathways and PPIs

The kinome annotation in KinBase provides a starting point for investigating protein phosphorylation networks in mammals. Given the experimentally validated kinase-specific phosphorylation sites, the intracellular phosphorylation networks between kinases and substrates could be reconstructed. In addition to the kinase–substrate phosphorylations, this update has integrated the information of metabolic pathways and PPIs to implement the network analysis for a group of interested genes/proteins. In this work, a public network visualization software, Cytoscape (41), was used to design a user interface for exploring the protein kinase–substrate phosphorylation networks, as well as the associated metabolic pathways and PPIs. The information of metabolic pathways associated with human, mouse and rat was referred to the annotations in KEGG (42). For the information of experimentally verified physical interactions, >10 PPI databases (as listed in Supplementary Table S2) have been integrated. In addition to physical interactions, the STRING database also consists of predicted functional associations (co-regulation in curated pathway, co-occurrence in literature abstracts, mRNA co-expression and genomic context) with confidence scores between proteins (43).

To make the construction of phosphorylation networks feasible, a graph theory has been adopted to formalize the networks between kinases and substrates, which were based on a KEGG pathway map. As presented in Supplementary Figure S2, the intracellular protein phosphorylation networks were visualized as a directed and cyclic graph *G* = (*V*, *E*), where *x*, *y* ∈ *V* and (*x*, *y*) ∈ *E*. Let *x* and *y* represented kinase and substrate proteins, respectively, and (*x*, *y*) ∈ *E* represented a relation of protein phosphorylation when kinase *x* phosphorylated substrate *y*. However, the intracellular phosphorylation networks not only contained the kinase cascades or kinase–substrate phosphorylations but also PPIs or protein complex. Thus, the (*x*, *y*) could stand for a relation of PPI between two proteins *x* and *y*. In this work, *V* referred to all proteins of human, mouse and rat, and *E* referred to all experimentally verified relations in RegPhos including experimental kinase–substrate phosphorylations and experimental PPIs. Users are allowed to input a group of proteins/genes into RegPhos 2.0, and the system efficiently returns the protein phosphorylation networks associated with three network models with PPIs, subcellular localization and metabolic pathway.

### Network investigation combining quantitative time-resolved phosphoproteome data

Phosphorylation cascades mediated by protein kinases regulate signaling transduction and cellular function. Accumulated literature has reported that dynamic change of global phosphorylation induces significant cellular responses (44–46). To investigate the cross talk in phosphorylation networks, the quantitative time-coursed phosphoproteomic data were integrated manually from the research articles containing LC-MS/MS analysis. A previous work has applied a general mass spectrometric technology for identification and quantitation of phosphorylation sites after stimulating HeLa cells with epidermal growth factor (EGF) and recorded in the Phosida database (44). The dynamic phosphoproteome provided a missing link in a global view of cellular processes. Cao *et al.* have proposed a quantitative time-resolved phosphoproteomic analysis for F*cε*RI-mediated mast cell signaling through a time-course of F*c*R stimulation in 0 s, 10 s, 30 s and 1–10 min (45). Additionally, newly discovered phosphorylation event and sites across a time-course of receptor stimulation also provided the direct observation in stable isotope labeling of amino acids in cell culture-labeled Zap-70 null and Zap-70 reconstituted T cells (46). Development of systematic method for elucidating dynamic phosphorylation events is therefore crucial for a full understanding of cellular behavior. As presented in Figure 1, the time-coursed phosphoproteome data, stimulated with different time points, were used to investigate the expression behavior of the discovered phosphorylation networks associated with subcellular localization. Pearson correlation coefficient was adopted to measure the similarity of two expression profiles. Based on the *k*-means clustering method, the expression profiles of phosphopeptides derived from LC-MS/MS analysis could be roughly categorized into five groups, membrane-associated, cytoplasmic upstream, cytoplasmic, cytoplasmic downstream and nuclear phosphopeptides. After the construction of protein kinase–substrate phosphorylation networks, the clustered phosphopeptides were mapped to the network members for verifying the expression behavior of intracellular signaling networks, starting from tyrosine receptor kinases to nuclear kinases or TFs.

### Differential expression analysis of kinase and substrate genes in 39 cancer types

It has been estimated that around half of kinome is disease- or cancer-related by chromosomal mapping. Additionally, receptor tyrosine kinases are the hallmark of a cancer cell and are involved in the prognosis of the most common forms of cancer. (47). To provide a disease analysis for kinases and phosphoproteins, the annotation involving diseases and drugs in KEGG (48) has been integrated in this update. Gene expression profiling has been demonstrated as a practical means to reveal cancer-specific signatures and could identify membrane proteins that are related to cancer progression (49). The overexpressed receptor kinases are becoming increasingly important in developing therapeutic target for cancers. With reference to the comprehensive collection of gene expression data in GEO database (50), the microarray experiments associated with cancers were used to explore the expression profile of the genes coding for kinases and their substrate proteins in various tumor cells. As listed in Supplementary Table S3, a total of 30 experiment series containing 39 cancer types from Affymetrix Human Genome U133 Plus 2.0 Array (GPL570), consisting of 54,675 probe set for >47,000 transcripts, were integrated in this work. All of the integrated samples were normalized by Robust Multichip Average (RMA) algorithm (51). RMA normalization was performed by the ‘justRMA()’ function of Bioconductor Affy package in R program language using raw data (Affymatrix CEL file). Then, the fold change values of genes between cancer and normal cells were log2 transformed for identifying the upregulated (fold change value > 1) and downregulated (fold change value <−1) genes in 39 cancer types (*P* < 0.01).

## Data content and utility

### Data statistics in RegPhos 2.0

In this update, all of the data used in construction of intracellular phosphorylation networks were experimentally validated. After the removal of data redundancy and inconsistency, as presented in Table 1, totally 66,301, 41,716 and 3754 experimentally confirmed phosphorylation sites are annotated on 10,257 human, 7306 mouse and 1203 rat phosphoproteins (substrates), respectively. All of the experimental phosphorylation sites were supported by >15,000 research articles. Among the phosphorylation sites integrated in RegPhos 2.0, only 7091 human substrate sites (∼10%) have the annotation of catalytic kinases, which results in 4036 kinase–substrate phosphorylation pairs. Also, merely 1062 mouse and 423 rat phosphorylation sites have the annotation of catalytic kinases, leading to 684 and 270 kinase–substrate phosphorylation pairs, respectively. According to the annotations of kinase families in KinBase, the data statistics of kinase-specific phosphorylation sites in different species, as well as the sequence logo of kinase substrate motifs, were presented in Supplementary Table S4. For instance, the protein kinase A (PKA) family, consisting of three kinase members, phosphorylated 392 substrate sites in 194 human proteins, 112 substrate sites in 59 mouse proteins and 76 substrate sites in 38 rat proteins. These experimentally verified kinase–substrate pairs are the main data for reconstructing the intracellular phosphorylation networks in mammals.

**Table 1.** Data statistics of the experimentally verified kinases, phosphorylation sites, substrate proteins and kinase-associated interactions in human, mouse and rat

Species	Human	Mouse	Rat
Number of kinases	518	540	306
Number of kinase families	221	195	159
Number of phosphorylated proteins (substrates)	10,257	7306	1203
Number of phosphorylation sites	66,301	41,716	3754
Number of phosphorylation sites with catalytic kinase	7091	1062	423
Number of kinase–substrate phosphorylation pairs	4036	684	270
Number of kinase-interacting proteins	12,910	5810	1442
Number of kinase–protein interactions	76,855	13,122	2655
Supported literatures	10,976	3089	1864

To provide a more comprehensive network analysis, the interactions between kinases and other proteins are incorporated with kinase substrate motifs to identify the potential kinases for the remaining phosphorylation sites without the annotation of catalytic kinases. According to the information of physical interactions and functional associations integrated in RegPhos 2.0, there are 12,910 proteins interacting with 518 human kinases, which results in 76,855 kinase–protein interactions. In mouse interaction data, there are 13,122 kinase–protein interactions between 540 kinases and 5810 mouse proteins, while 2655 kinase–protein interactions were annotated between 306 kinases and 1442 proteins in rat.

### Web interface of exploring protein phosphorylation networks

This update extends RegPhos to be an informative resource for exploring the protein kinase–substrate phosphorylation networks in mammals. To facilitate the access to RegPhos, the web interface has been redesigned and enhanced for users to efficiently browse and search for interested kinases as well as their substrate proteins. The typical query for a kinase includes basic protein information, gene expression profile in 39 cancers, summary table of substrate proteins and network analysis between kinase and their substrates. As presented in Supplementary Figure S3, the basic information about a kinase or substrate includes protein function, subcellular localization, protein domains and tertiary structures. Additionally, the RegPhos provides the expression profile of a gene coding for the interested kinase or substrate in 39 cancers. A summary table including substrate proteins as well as the number of phosphorylation sites was provided for each kinase. Then, users could investigate the phosphorylation network among the interested kinase and the selected substrate proteins, associated with the information of PPI, subcellular localization and metabolic pathway.

**Figure 2.**
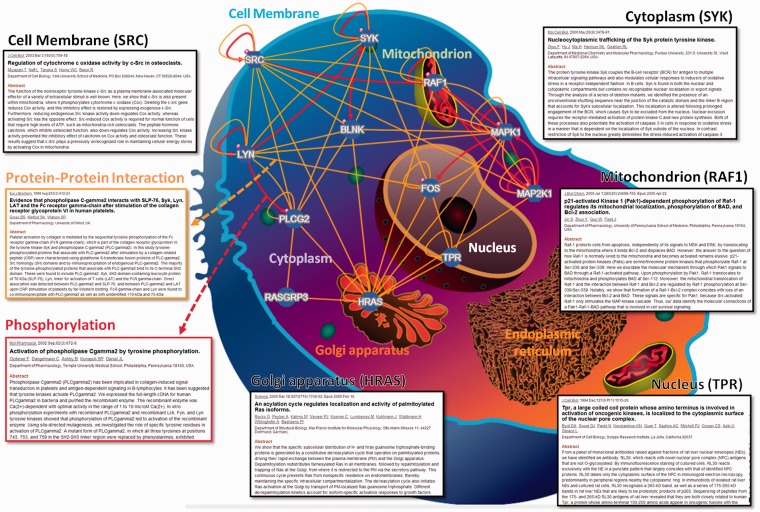
A case study of network analysis with the information of protein subcellular localization.

In RegPhos 2.0, three network models were provided to explore the intracellular kinase–substrate phosphorylation networks. As shown in Supplementary Figure S4, the first model is ‘Network with protein–protein interaction’. Because users input a group of proteins, the RegPhos identifies the kinases and phosphoproteins for the inputted proteins and connects them with the information of kinase–substrate phosphorylations and PPIs. This is an interactive interface for users to move the nodes arbitrarily and click on the nodes to access the information about kinase or substrate in detail. Additionally, users can click on the edges to access the information about phosphorylation or PPI. The second model in network analysis is ‘Network with subcellular localization’. In eukaryotic cell, proteins always work together and locate in the same subcellular localization to perform particular functions (52). Therefore, understanding the localization of every protein is important for investigating its interactions with other molecules and for elucidating its biological function. In this update, the information of protein subcellular localization was used to construct the intracellular phosphorylation network starting from a receptor or membrane-associated proteins to TFs or proteins in nucleus. As presented in Figure 2, the inputted proteins was located in specific cellular components, such as cell membrane, cytoplasm, mitochondrion, Golgi apparatus, endoplasmic reticulum and nucleus, with reference to the annotations of protein subcellular localization obtained from external databases. For instance, the tyrosine-protein kinase Lyn (LYN) and proto-oncogene tyrosine-protein kinase Src (SRC), which contain a protein kinase domain playing an important role in membrane-associated localization (53, 54), are located closely to cell membrane. The GTPase H-Ras (HRAS) can shuttle between plasma membrane and golgi apparatus (55). Spleen tyrosine kinase (SYK) is found in both the nuclear and cytoplasmic compartments but contains no recognizable nuclear localization or export signals (56). The phosphorylation of RAF proto-oncogene serine/threo-nine-protein kinase (RAF1) is required for its mitochondrial localization (57). Nucleoprotein TPR is involved in activation of oncogenic kinases and is localized to the cytoplasmic surface of the nuclear pore (58). Following induction of cell growth factor, the proto-oncogene c-Fos (FOS) firstly localizes to endoplasmic reticulum and later to the nucleus (59). Therefore, the network combining subcellular location, PPI and literature mining can help us to understand the biological significance and regulatory function of kinase-to-substrate in phosphorylation cascade.

With the importance of protein phosphorylation in regulating metabolic pathways and signal transduction, this work has incorporated Cytoscape program with public pathway maps obtained from KEGG to implement the third model of network analysis. As presented in Supplementary Figure S5, the inputted proteins are mapped to the items on a KEGG pathway map, which indicates how many proteins are involved in BCR signaling pathway. However, some of the inputted proteins could not be matched to the items but have connections with the mapped proteins on a KEGG pathway map. For instance, the SRC, which was not reported to be involved in classical BCR signaling pathway, has connections with the matched proteins, such as SYK, LYN, BLNK, RAF1, HRAS and MAPK kinases. This investigation indicated that the SRC has a strong connectivity with BCR signaling.

### A case study of the discovered networks associated with BCR signaling

**Figure 3.**
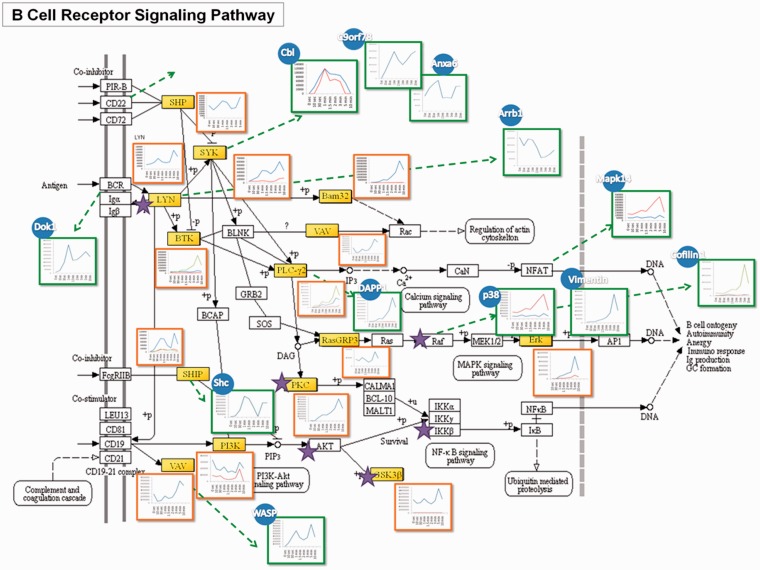
A case study of the RegPhos-discovered phosphorylation networks involved in BCR signaling pathway. The phosphoproteome change in response to F*cε*RI-mediated mast cell activated with F*c*R at 9 time points was used to validate the phosphorylation profile of the proteins in the discovered phosphorylation networks.

A published tyrosine phosphoproteomic data from F*cε*RI-mediated mast cell signaling activated by F*c*R at 9 time points (45) has been analyzed and functioned as a model study to demonstrate the feasibility of the RegPhos 2.0, which not only attempted to comprehensively illustrate the profile of the signaling cascade but also the involved protein-interaction network. Take BCR signaling as an example, as shown in Figure 3, mapping the phosphorylation data (containing 125 tyrosine phosphoprotein) to the BCR signaling pathway from KEGG, the identified molecules were highlighted in yellow, and the kinases (i.e. Lyn, Btk, etc.) were marked with a star. Many central molecules, such as Lyn, Syk and Btk, were identified. The trend of phosphorylation level after activation was displayed to reveal the site-specific phosphorylation change at different time point (shaped in red square).

Aside from the molecules in conventional BCR signaling, through the PPI, many other protein phosphorylations in response to activation can be linked to this pathway. Those molecules may also directly involve in this signaling cascades or through interactions between proteins, which can be revealed by phosphoproteome and bioinformatic analysis. To address this issue, phosphorylation data were inputted to generate a protein interaction network of the putative BCR-medicated signaling cascade using database that integrates experimentally verified interactions from different sources. The interacting proteins were illustrated in blue circles and their expression patterns were showed in green squares. Cbl, C9orf78 and Anxa6 can be linked to this pathway via the interaction with Syk (60, 61), while Arrb1 was known to bind with Lyn (62). Dok1 has the interaction with BCR (63). One notable feature of this network was that these interacting proteins of upstream molecules showed higher phosphorylation level at the early time point, suggesting their involvement in the early stage of activation. Shc was reported to physically interact with SHIP and increase its activity (64). Its phosphorylation pattern showed the same trend as that of SHIP. Phosphorylation of DAPP1 showed the same trend with that of its interacting protein, PLCγ2 (65), while the phosphorylation pattern of WASP was similar to that of VAV, indicating their interaction during the activation. Moreover, phosphorylation of Mapk14 has been reported to be able to phosphorylate nuclear factor of activated T-cells (NFAT) members (66). Although the phosphorylaiton of NFAT on tyrosine residues was not identified here, through observing its interacting protein, Mapk14, the activation of the NFAT pathway was confirmed. Erk was found to have enhanced phosphorylation at 3–5 min, which is consistent with its upstream molecule, RasGRB3. The phosphorylation pattern of the interacting protein of Raf, p38 and Vimentin also showed similar pattern. CREB1 is one of the downstream molecules of p38. Cofilin 1 that has been known to have a putative CREB1-binding site was considered to involve in the same pathway. The phosphorylation profile of Cofilin 1 was identical to those in Erk pathway, suggesting its participation in this pathway. Based on the coordinate grouping by PPI, deciphering the complex network of signaling events and feedback loops will be important for understanding the underlying mechanisms of controlling cell functions.

### Investigation of phosphorylation-associated biomarkers in cancers

Owing to the difficulty of obtaining the protein expression evidence associated with cancers from available databases, this work has integrated the gene expression data from GEO database. A total of 30 microarray experiment series containing 39 cancer types have been used to investigate the expression profile of 528 human kinase genes in tumor cells. Supplementary Table S5 listed the discriminatively expressed kinase genes in 39 cancer types. According to the expression profile of microarray experiment (GSE10780) involving 42 samples in invasive ductal breast carcinoma (IDC) versus 143 samples in normal breast tissues, 11 upregulated and 7 downregulated kinase genes were identified in breast cancer. As shown in Supplementary Figure S6, three upregulated kinases (ERBB2, MAPK1 and MAP2K2) and two downregulated kinases (EGFR and RAF1) were involved in ERBB signaling, which controls mammosphere formation in human breast cancer (67). Interestingly, the ELK1, phosphorylated by MAPK1 and associated with cell survival in breast tumor (68), also has a relatively higher expression in breast cancer. Extracting the similar expression level, based on the same phosphorylation signaling cascade by relationship between kinase and substrate, suggests that ELK1 may be an important regulator in ErbB2 pathway. On the other hand, phosphorylation of ELK1 is positively regulated by EGFR expression and phosphorylation (69). However, EGFR whose expression and association that has been identified in breast cancer (70, 71) contains a relatively low expression in this microarray experiment. According to this microarray experiment involving IDC, the lower expression of RAF1 and MYC might correlate with the decreased expression of EGFR. The mechanism for regulating transcription or phosphorylation of ELK1 via ERBB2 and EGFR pathway needs to be further clarified. Consequently, the network analysis in RegPhos 2.0 combining the gene expression profile and PPI could provide a preliminary investigation of potential biomarkers in cancers.

## Conclusion

Owing to the importance of protein phosphorylation in regulating a variety of intracellular processes, this update aims to provide a more comprehensive view of intracellular signaling networks by integrating the information of metabolic pathways and PPIs. The RegPhos 2.0 not only enhances the data content in human but also investigates kinase–substrate phosphorylation networks in mouse and rat. The quantitative time-resolved phosphoproteome profiling in mast cells has been used to demonstrate that RegPhos could identify novel network members that have consistent expression behavior with known proteins involved in BCR signaling pathway. Additionally, the integration of 30 microarray experiments provides a prospective analysis for identifying phosphorylation-associated biomarkers in 39 cancers. The differentially expressed kinase and substrate genes in a specific cancer might be the potential targets for drug design. An exhaustive comparison of data features and web functions between RegPhos 1.0 and 2.0 was listed in Table 2. In the future, the growth of RegPhos is expected as the availability of data increases in resources related to protein phosphorylation. To provide more adequate information needed for functional analysis, the descriptions associated with the biological function of phosphorylation sites will be extracted with increased precision from research articles by using an enhanced information retrieval system. Additionally, a recent study (72) has extracted 3D-signature motifs from experimentally verified phosphorylation sites with 3D structures available in PDB. We can envision that RegPhos can be greatly improved in prospective works by applying the 3D-signature motifs to investigate the phosphorylation sites on protein tertiary structures.

**Table 2.** The comparison of data features and web functions between RegPhos 1.0 and 2.0

Features	RegPhos 1.0	RegPhos 2.0
Species	Human	Human, mouse and rat
Protein entry	UniProtKB/Swiss-Prot (release 55)	UniProtKB release 2013–04
External phosphorylation resource	UniProtKB/Swiss-Prot, Phospho.ELM, PHOSIDA and HPRD	UniProtKB/Swiss-Prot, Phospho.ELM, PHOSIDA, HPRD, PhosphoSitePlus and sysPTM
Manual literature survey	None	More than 500 kinase-specific phosphopeptides from ∼200 articles
Computational annotation of catalytic kinases for *in vivo* phosphorylation sites	68 kinase groups	Over 100 kinase groups
Data content for network construction	Experimental kinase–substrate phosphorylations and PPI	Experimental kinase–substrate phosphorylations, PPIs and KEGG metabolic pathways
Network analysis	Network with PPI	Network with PPI, Network with protein subcellular localization and Network with metabolic pathway map
Network visualization	PHP GD library	PHP GD library and Cytoscape package
Network verification	Time-coursed gene expression profile	Manually curated quantitative time-resolved phosphoproteome data obtained from LC-MS/MS analysis
3D structure of phosphorylation sites	None	PDB and Jmol viewer
Protein domain	InterPro	InterPro and InterProScan
PPI	DIP, MINT, IntAct, HPRD and STRING	Over 10 public PPI resources
Cancer analysis	None	Kinase and substrate gene expression profile in 39 cancers
Disease information	None	KEGG Disease database
Download	None	All of the kinase–substrate phosphorylations could be downloaded from website

## Availability

The data content in RegPhos will be maintained and updated quarterly by continuously surveying the public resources and research articles. Also, the microarray expression data involved in human diseases will be semiannually collected from Gene Expression Omnibus (GEO). The resource is now freely accessed online at http://csb.cse.yzu.edu.tw/RegPhos2/. All of the experimentally verified phosphorylation sites and kinase–substrate interactions could be downloaded in the text format.

## Supplementary data

Supplementary Data are available at *Database* Online.

## Funding

This work was supported by Ministry of Science and Technology, Taiwan (101-2628-E-155-002-MY2, 101-2311-B-009-003-MY3, 102-2911-I-009-101 and 100-2628-M-001-003-MY4); Thematic Research Program of Academia Sinica, Taiwan (AS-102-TP-A03); the Veterans General Hospitals and University System of Taiwan (VGHUST) (VGHUST103-G5-1-2). Funding for open access charge: AS-102-TP-A03.

*Conflict of interest*. None declared.
